# Author Correction: Calcium-permeable channelrhodopsins for the photocontrol of calcium signalling

**DOI:** 10.1038/s41467-024-48356-4

**Published:** 2024-05-10

**Authors:** Rodrigo G. Fernandez Lahore, Niccolò P. Pampaloni, Enrico Schiewer, M.-Marcel Heim, Linda Tillert, Johannes Vierock, Johannes Oppermann, Jakob Walther, Dietmar Schmitz, David Owald, Andrew J. R. Plested, Benjamin R. Rost, Peter Hegemann

**Affiliations:** 1 Institute of Biology, Experimental Biophysics, Humboldt-Universität zu Berlin, Berlin, Germany; 2 Molecular Neuroscience and Biophysics, Leibniz-Institut für Molekulare Pharmakologie, Berlin, Germany; 3 Institute of Biology, Cellular Biophysics, Humboldt-Universität zu Berlin, Berlin, Germany; 4 Institute of Neurophysiology, Charité – Universitätsmedizin Berlin, Berlin, Germany; 5 Neuroscience Research Center, Charité – Universitätsmedizin Berlin, Berlin, Germany; 6 Department of Neurology with Experimental Neurology, Charité – Universitätsmedizin Berlin, Berlin, Germany; 7 German Center for Neurodegenerative Diseases (DZNE), Berlin, Germany

**Keywords:** Optogenetics, Permeation and transport, Cellular neuroscience

Correction to: *Nature Communications* 10.1038/s41467-022-35373-4, published online 21 December 2022

Since the publication of this work, Enrico Schiewer has changed their name from Enrico Peter. This has now been amended in the HTML and PDF versions of the article.

