# Supplementary material for: Calcium-permeable channelrhodopsins for the photocontrol of calcium signalling
Source: Nat Commun. 2022 Dec 21;13:7844. doi: 10.1038/s41467-022-35373-4 (PMC9772239; doi:10.1038/s41467-022-35373-4)
Supplement: Supplementary file 1 — Supplementary Information [file 41467_2022_35373_MOESM1_ESM.pdf]

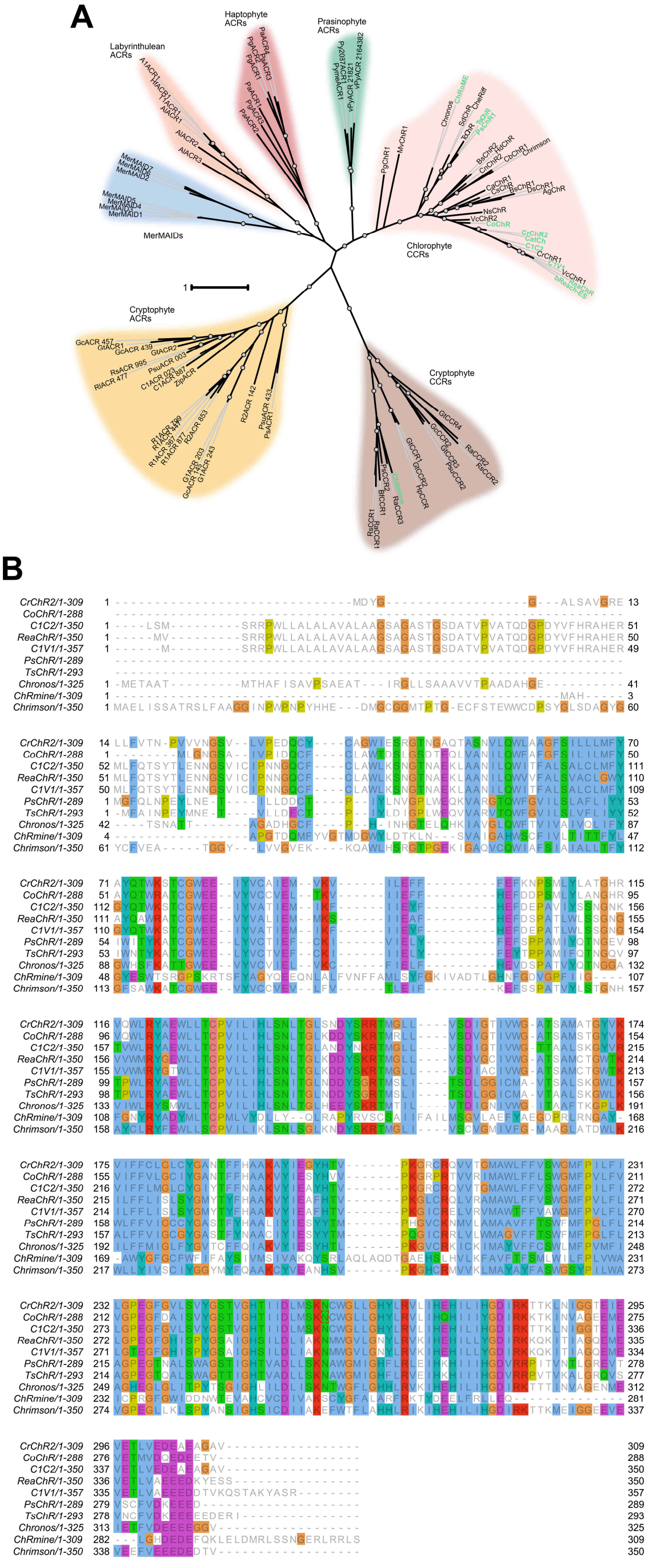

Supplementary Fig. 1: Phylogeny and sequence alignment of selected CCRs. A) Unrooted phylogenetic tree of the channelrhodopsin family. Scale bar indicates the average number of residue substitutions per site. Gray circles indicate ultrafast bootstrap support values >95%. Channelrhodopsins analyzed in this publication (Fig. 1C, D) have been marked in green. B) Sequence alignment of selected channelrhodopsins. Residues are colored according to the ClustalX color scheme using Jalview. GenBank/PDB accession numbers: *Cr*ChR2 (AF508966), *Co*ChR1 (AHH02107), C1C2 (PDB: 3UG9, http://doi.org/10.2210/pdb3UG9/pdb), ReaChR (KF448069.1), C1V1 (KF448069.1), *Ps*ChR (JX983143.1), *Ts*ChR (AHH02155), Chronos (KF992040.1), ChRmine (QDS02893), Chrimson (AHH02126). Abbreviations: ACRs, Anion-conducting channelrhodopsins; CCRs, Cation-conducting channelrhodopsins; MerMAIDs, Metagenomically discovered marine anion-conducting and intensely desensitizing channelrhodopsins.

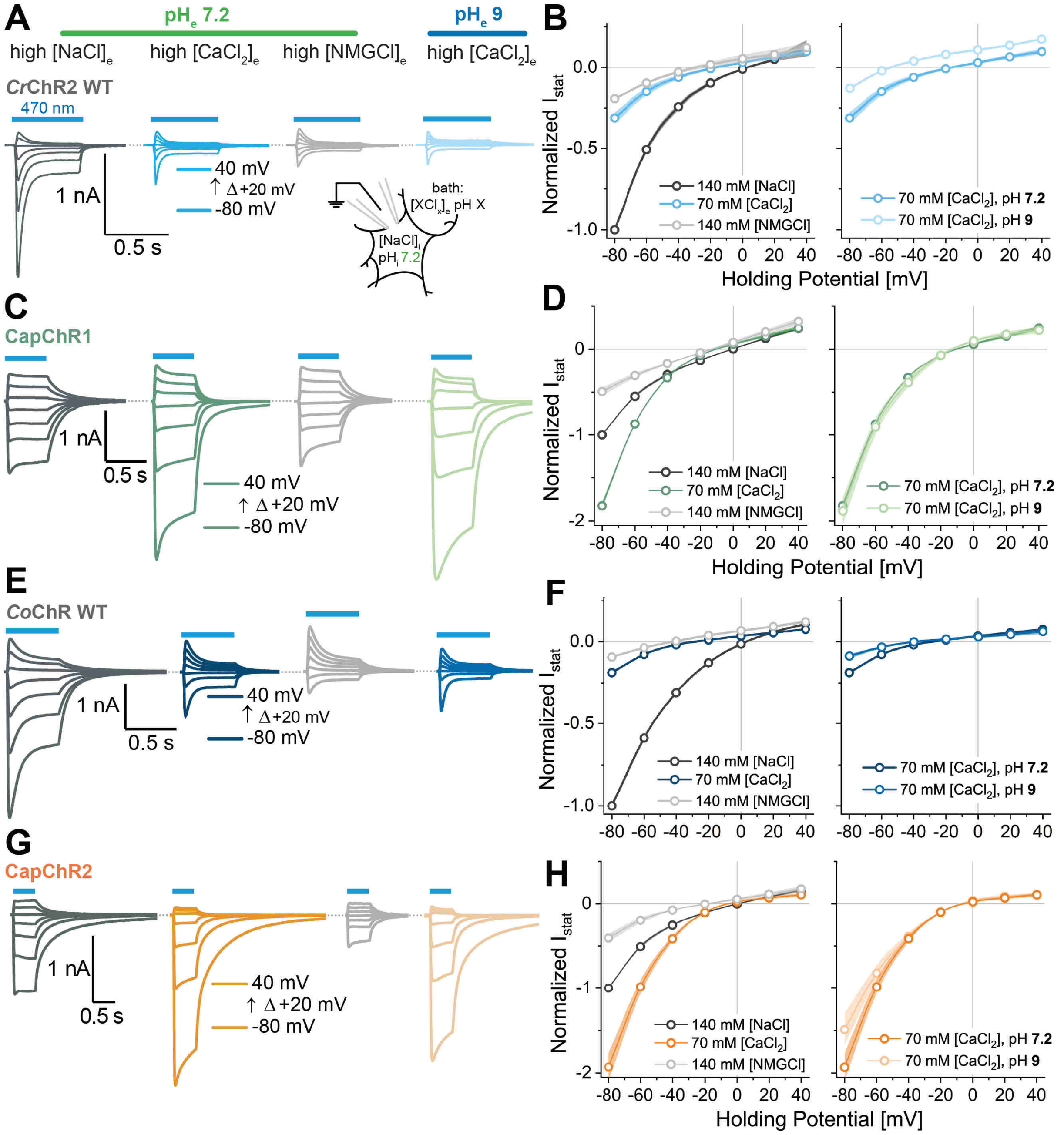

Supplementary Fig. 2: Electrophysiological characterization of C2, *Co*ChR CapChR1 and CapChR2. A, C, E and G) Representative photocurrents traces of C2 (light blue), CapChR1 (green), *Co*ChR WT (dark blue) and CapChR2 (orange) respectively, recorded from -80 to +40 mV in 20 mV steps in the denoted buffer conditions in ND7/23 cells (blue bar: illumination with saturating, 470 nm light; ~1.9 mW/mm²). B, D, F, H) I-V relationships with estimated reversal potentials (inlets) for ChRs variants at the designated ionic conditions (Mean ± S.E.M., n=3-14). Dots represent mean values and shadows the S.E.M. n=X biologically independent cells.

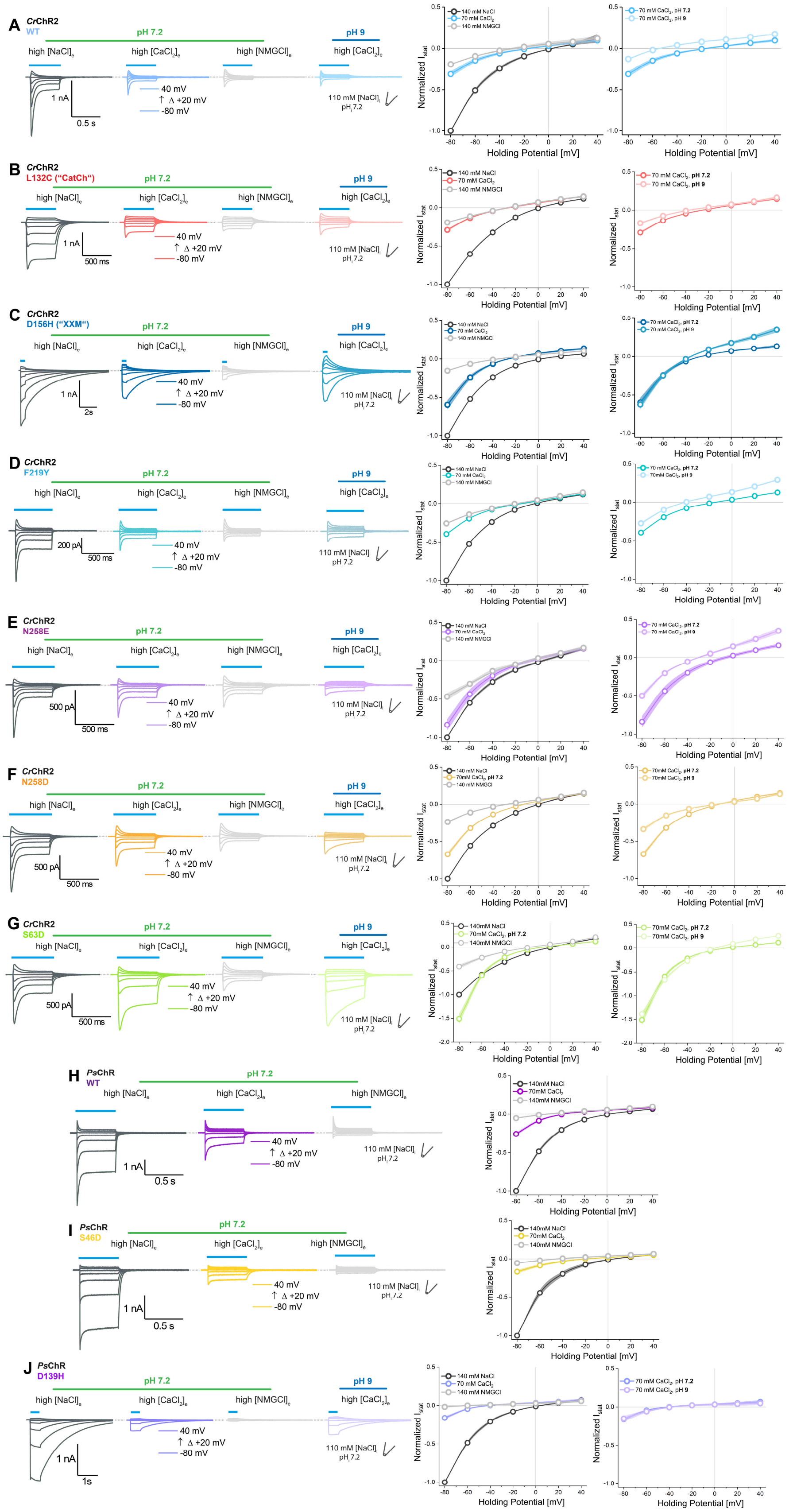

Supplementary Fig. 3: Electrophysiological characterization of *Cr*ChR2 and *Ps*ChR variants. A-J) Left: Representative photocurrent traces of selected variants, recorded from -80 to +40 mV in 20 mV steps under the denoted buffer conditions in ND7/23 cells (blue bar: illumination with saturating, 470 nm light; ~1.9 mW/mm²). Right: I-V relationships for ChR variants at the designated ionic conditions (Mean ± S.E.M., n=3-11). Dots represent mean values and shadows the S.E.M. n=X biologically independent cells.

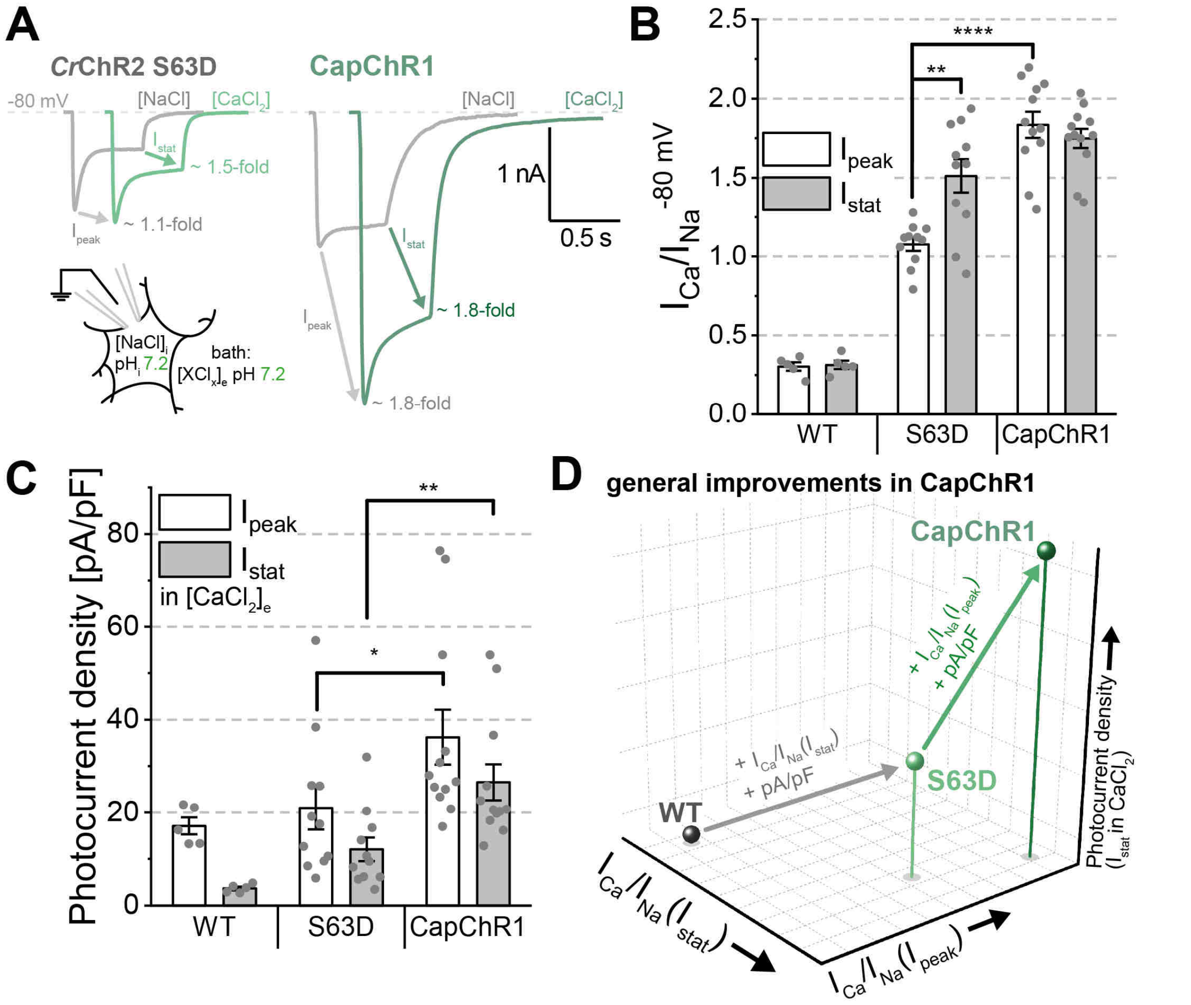

Supplementary Fig. 4: CapChR1 displays suppressed Na⁺-permeation at negative holding potentials. A) Representative photocurrent traces of *Cr*ChR2 S63D and CapChR1 at -80 mV in ND7/23 cells under the denoted buffer conditions. B) Stationary (I_{stat}) and peak (I_{peak}) I_{Ca}/I_{Na} of the denoted derivatives at -80 mV holding potential (Mean ± S.E.M.). C) Stationary and peak photocurrent densities of the denoted constructs at -80 mV holding potential (Mean ± S.E.M.). D) Visualization of the advantages provided by CapChR1, including increased I_{Ca}/I_{Na} for both peak and stationary photocurrents in conjunction with improved photocurrent densities. Within a mutant: paired, two-sided Wilcoxon-Mann-Whitney-Test; Between mutants: unpaired, two-sided Wilcoxon-Mann-Whitney-Test; *P ≤ 0.05, **P ≤ 0.01, ***P ≤ 0.001, ****P ≤ 0.0001. N for *Cr*ChR2 WT: n=5, S63D: n=11; CapChR1: n=12. n=X biologically independent cells. P-values in B): SD to SD comparison P=0.003; SD to CapChR1 comparison: 5.55 x 10^{-5}. P-values in C): I_{peak} comparison P=0.03; I_{stat} comparison P=0.003. n=X biologically independent cells.

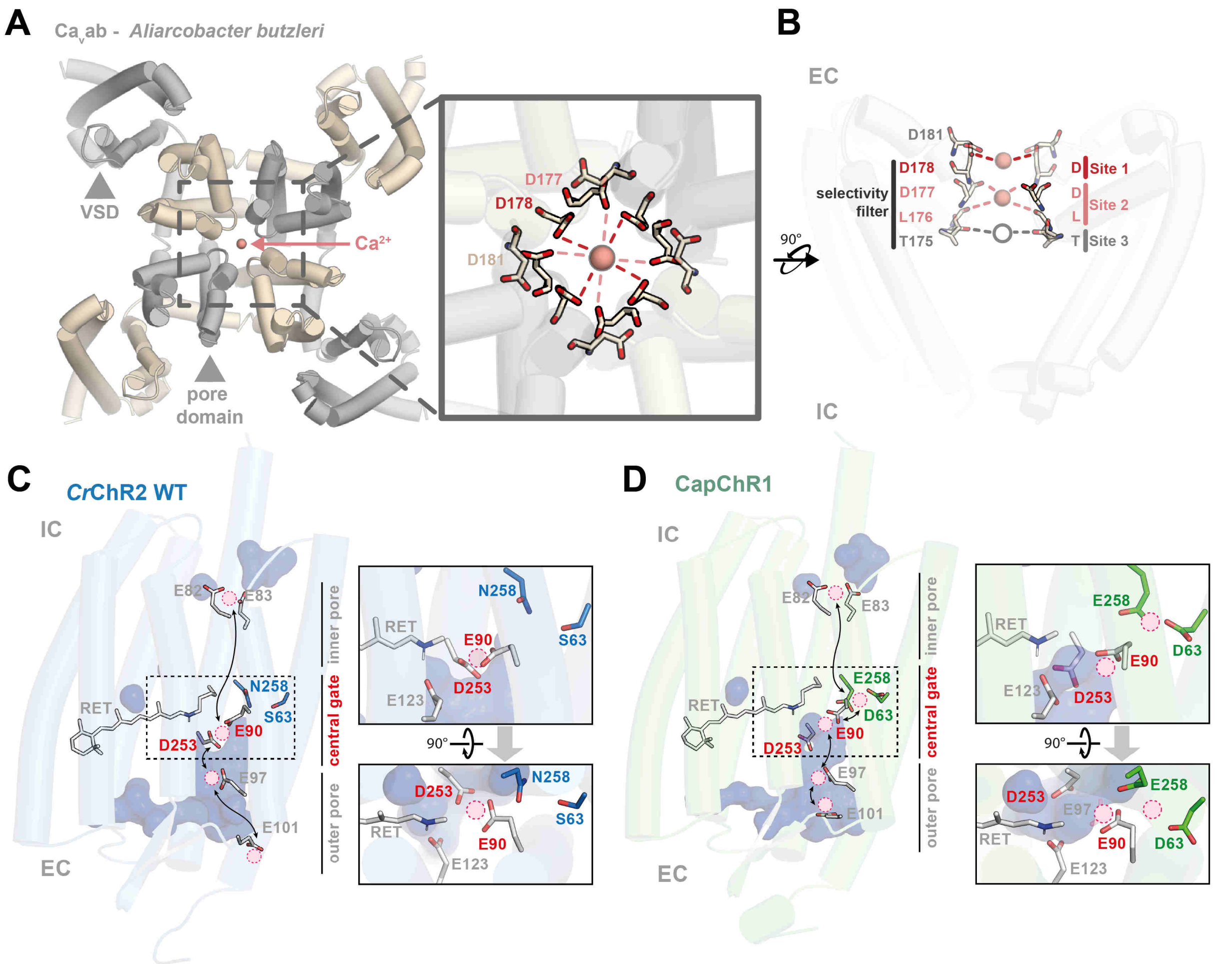

Supplementary Fig. 5: Structure of homotetrameric Ca$_v$Ab (PDB ID: 4MVQ, http://dx.doi.org/10.2210/pdb4mvq/pdb) and monomeric *Cr*ChR2 and derivative CapChR1. A) Top view of Ca$_v$Ab tetramer with voltage sensing domains (VSD) and pore domains with zoom-in (right) on bound calcium (rose). B) Side-view of the selectivity motif of Ca$_v$Ab, where carboxylic aspartates are mainly responsible for initial calcium binding and selectivity. In Ca$_v$Ab (parental protein is Na$_v$Ab, a sodium channel), E177, S178 and M181 were substituted with Asp to switch selectivity to Ca^{2+}. C) Side-view of *in silico* equilibrated *Cr*ChR2 WT and its derivative D) CapChR1 (based on PDB ID: 6EID, http://doi.org/10.2210/pdb6EID/pdb) with cofactor all-trans-retinal bound and side chains of amino acids possibly involved in cation binding and selectivity highlighted as licorice. The water-filled pores are shown as blue surfaces. Cation uptake on the extracellular side might be mediated by carboxylic residues E90, E97, E101 and D253, while Ca^{2+} selectivity is increased by introducing S63D and N258E at the central gate as additional negative charges (highlighted in zoom-ins as side- and top-views, mutated residues are colored). EC: extracellular, IC: intracellular.

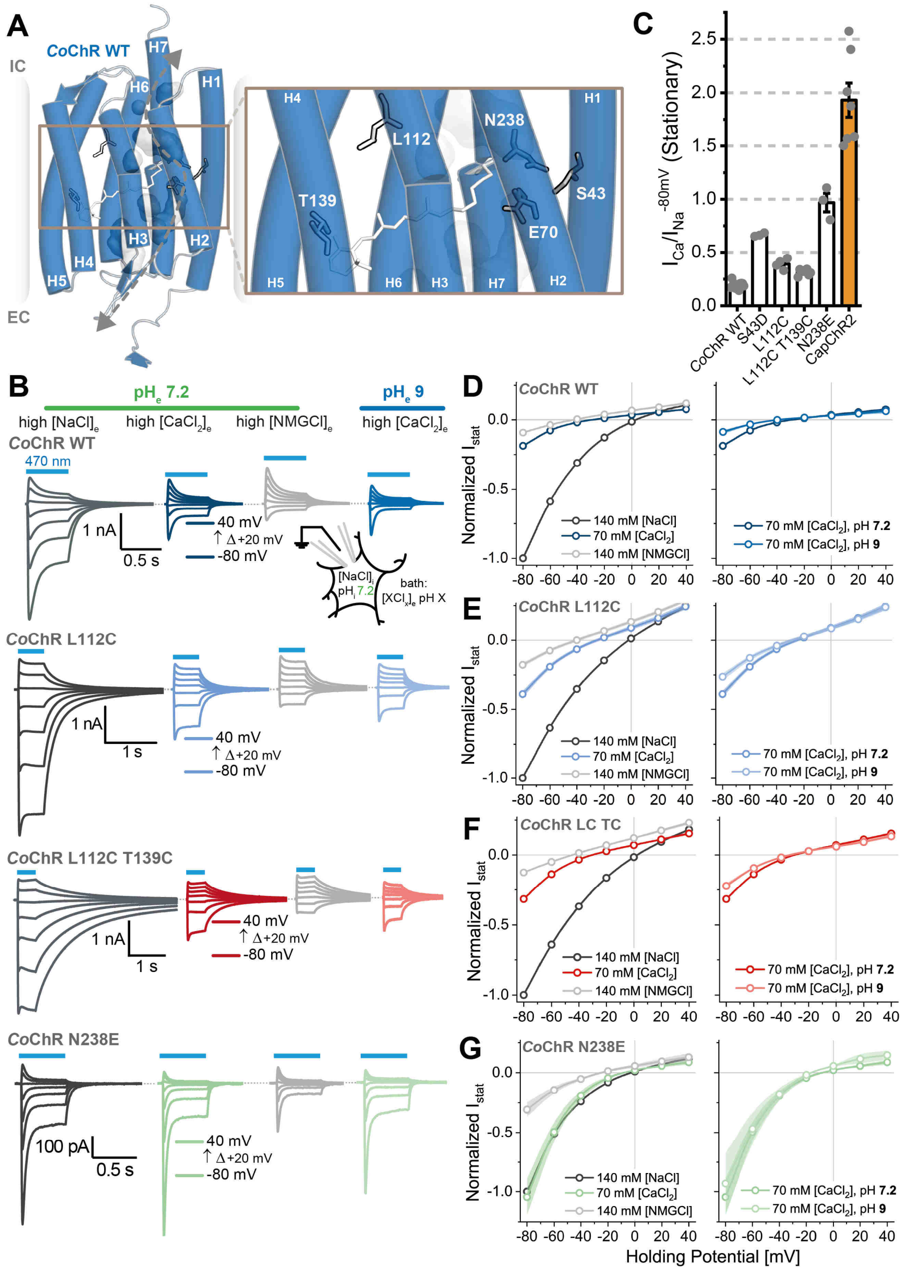

Supplementary Fig. 6: Calcium conductance of *Co*ChR WT, L112C, L112C T139C, S43D and N238E. A) *Co*ChR homology model depicting the location of the S43, L112, T139 and N238E residues in Helix 1, 3, 4 and 7 respectively (zoomed inlet, based on structure data, PDB ID: 6EID, http://doi.org/10.2210/pdb6EID/pdb). Grey mesh represents water accessibility according to MD simulations. Dark grey line represents the putative permeation pathway. B) Representative photocurrent traces of WT (dark blue), L112C (sky blue), and L112C T139C (red) and N238E (pale green) recorded from -80 to +40 mV in 20 mV steps in ND7/23 cells under the denoted buffer conditions (blue bar: illumination with saturating, 470 nm light; ~1.9 mW/mm²). C) Stationary I_{Ca}/I_{Na} of the denoted derivatives at -80 mV holding potential (Mean ± S.E.M.). Number of replicates WT/S43D/L112C/L112C T139C/N238E/CapChR2: n=12/3/4/8/3/7. D) I(E) relationships for the various mutants under varying extracellular conditions (shadows represent the S.E.M; normalized to high [NaCl] at -80 mV). Number of replicates in D-G) NaCl pH7.2/$CaCl_2$ pH7.2/NMGCl pH7.2/$CaCl_2$ pH9: (D) *Co*ChR WT n=12/12/10/3; (E) L112C n= 5/4/4/4, (F) LC TC n= 8/8/8/5, (G) N238E n= 3/3/3/3. n=X biologically independent cells. EC: extracellular, IC: intracellular.

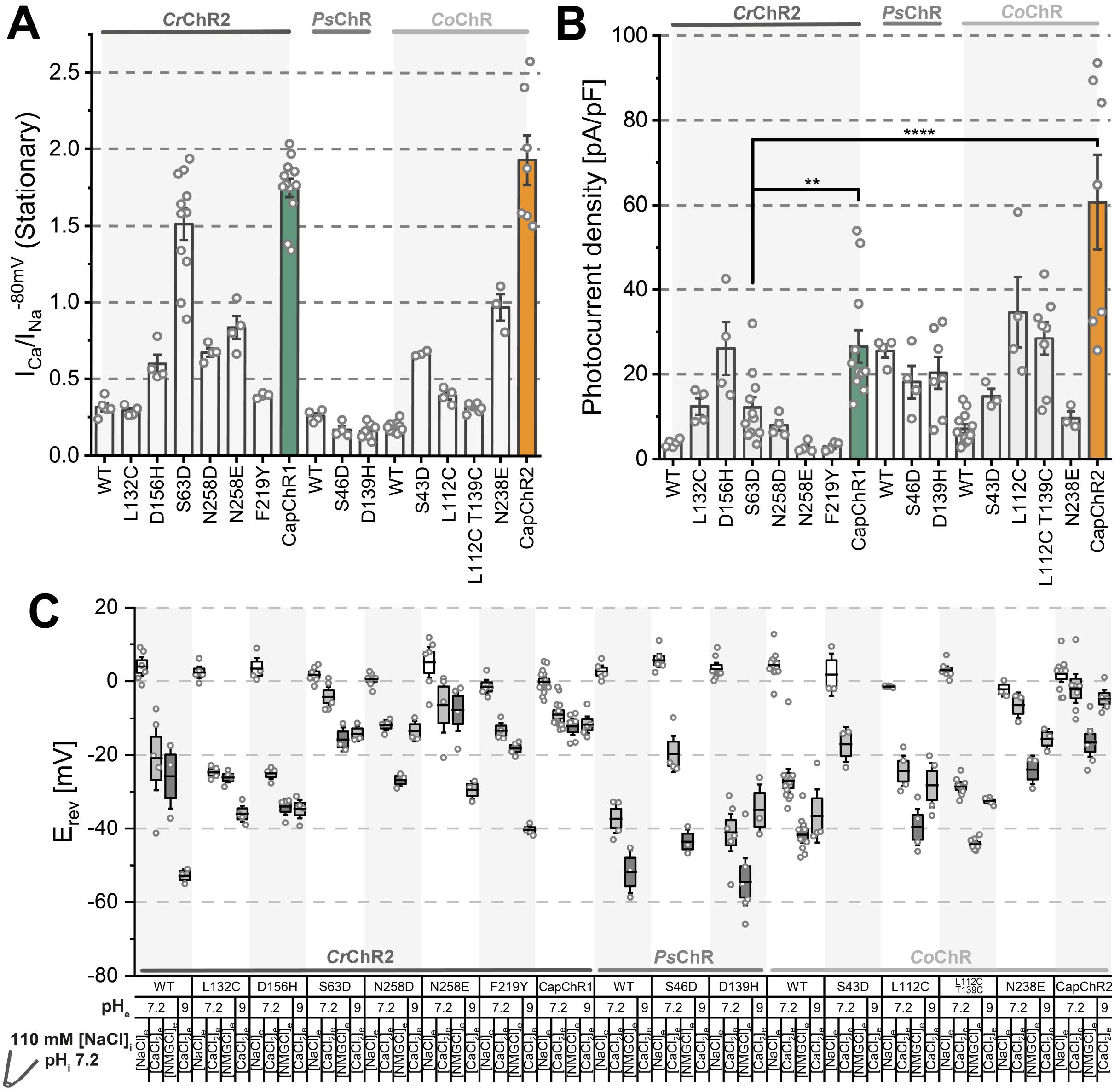

Supplementary Fig. 7: Electrophysiological characterization of *Cr*ChR2, *Co*ChR and *Ps*ChR variants. A) I_{Ca}/I_{Na} of the stationary photocurrent at pH 7.2 and -80 mV (Mean ± S.E.M., N=3-12, dots represent single measurements). B) Stationary photocurrent density of the denoted variants at 70 mM $[CaCl_2]_e$, pH 7.2 and -80 mV (Mean ± S.E.M., N=3-12, dots represent single measurements). C) Estimated reversal potentials under different ionic conditions for selected WT ChRs, single mutants and CapChR1 and 2 (Box middle line: Mean; Box outer edges ± S.E.M.; Box whiskers: 1.5 x S.E.M.; n=3-12, dots represent single measurements). Two-sided, unpaired Wilcoxon-Mann-Whitney-Test: *P ≤ 0.05, **P ≤ 0.01, ***P ≤ 0.001, ****P ≤ 0.0001. P values for comparison to *Cr*ChR2 S63D in B): CapChR1 P=0.003; CapChR2 P=8.05 x 10^{-4}. n=X biologically independent cells.

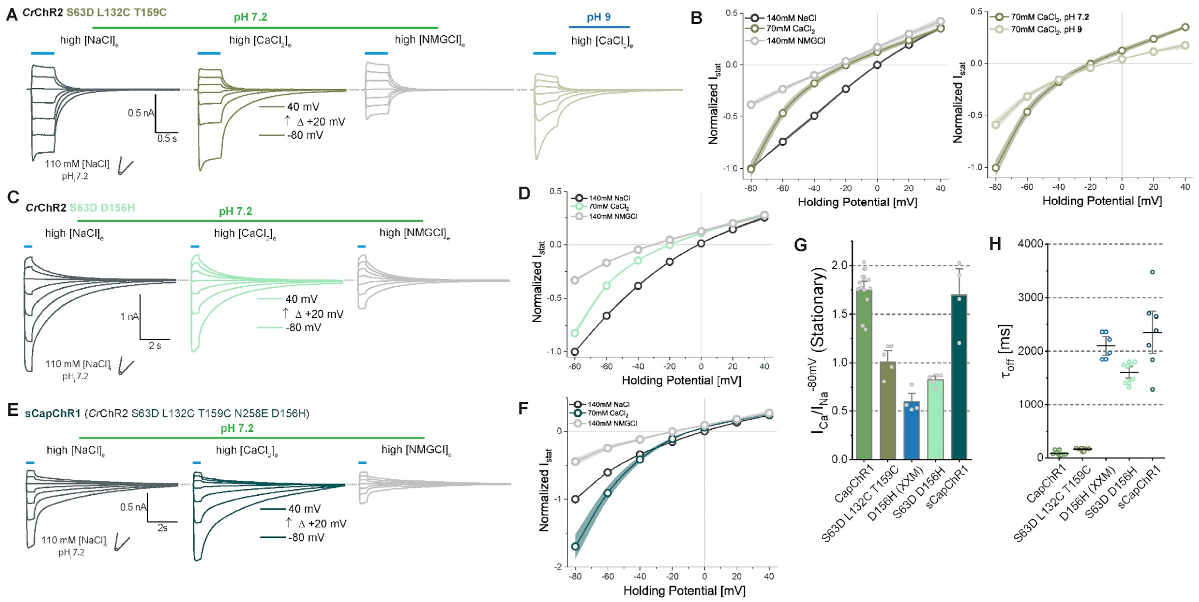

Supplementary Fig. 8: Electrophysiological characterization of *Cr*ChR2 and CapChR1 variants. A, C and E) Representative photocurrents traces of selected variants, recorded from -80 to +40 mV in 20 mV steps under the denoted buffer conditions in ND7/23 cells (blue bar: illumination with saturating, 470 nm light; ~1.9 mW/mm²). B, D and F) I-V relationships for ChR variants at the designated ionic conditions (Mean ± S.E.M., N=3-7). G) I_{Ca}/I_{Na} of the stationary photocurrent at pH 7.2 and -80 mV (Mean ± S.E.M., n=3-7, dots represent single measurements). H) Channel closure kinetics at 140 mM $[NaCl]_e$ and 2 mM $[CaCl_2]_e$ (τ_{off}). Lines and bars represent the mean ± S.E.M (n=4-7) and dots represent single measurements. sCapChR: slow calcium permeable channelrhodopsin 1. n=X biologically independent cells.

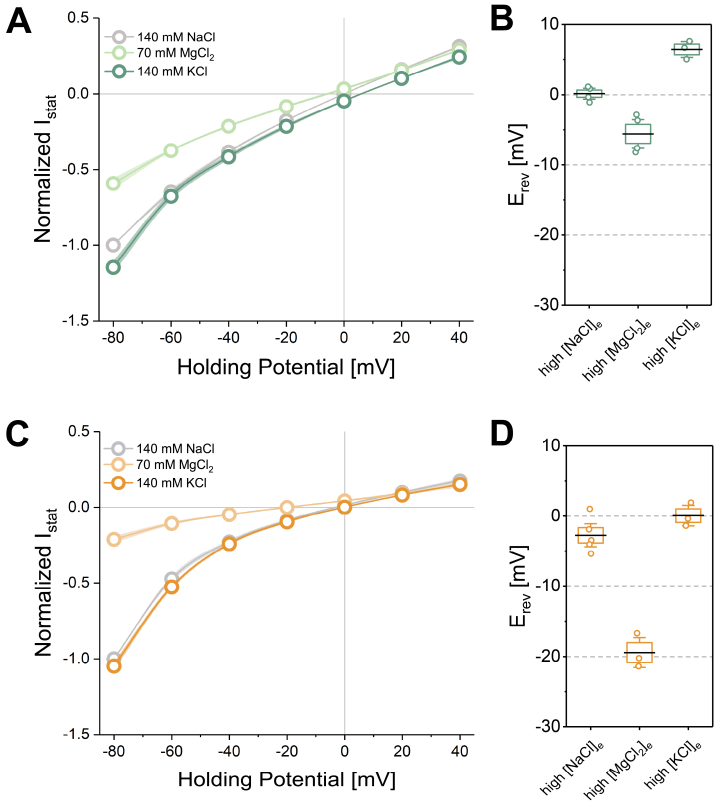

Supplementary Fig. 9: Potassium and Magnesium conductance in CapChR1 (green) and CapChR2 (orange). A and C) I-V relationships for CapChR1 and CapChR2 at the designated ionic conditions (Mean ± S.E.M., N=3-5). Dots represent mean values and shadows the S.E.M. B and D) Estimated reversal potentials (Box middle line: Mean, Box edges: ± S.E.M, Whiskers: ± SD, n=3-5, dots represent single measurements). n=X biologically independent cells.

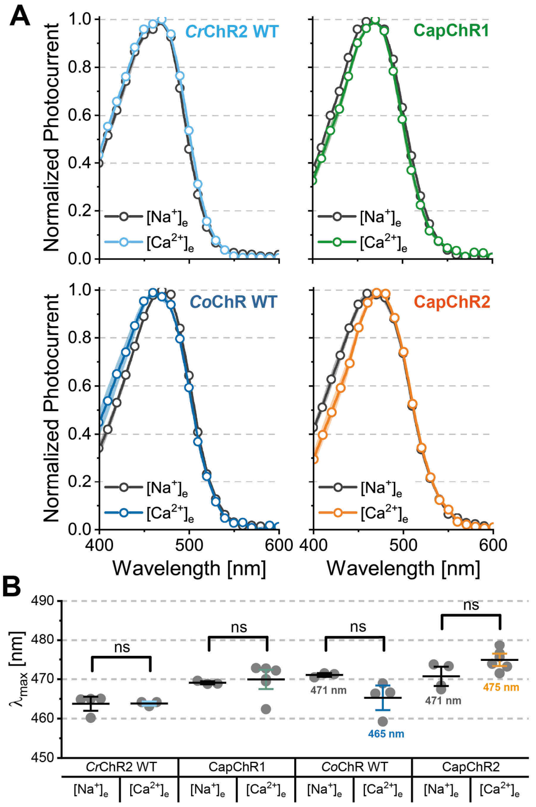

Supplementary Fig. 10: Action spectra of CapChRs and their parental ChRs. A) Normalized action spectra for *Cr*ChR2, CapChR1, *Co*ChR WT and CapChR2 at 140 mM $[NaCl]_e$ (dark gray) and 70 mM $[CaCl_2]_e$ (color) Dots represent mean values, shadows/bars the S.E.M. and lines the fitted data. B) Calculated absorption maxima under both buffer conditions for the four ChRs. Number of replicates Na^+/Ca^{2+}: for *Cr*ChR2 WT n=3/4; for *Co*ChR WT n=4/3; for CapChR1 n=6/3; for CapChR2 n=6/4. Two-sided, unpaired Wilcoxon-Mann-Whitney-Test: *P ≤ 0.05, **P ≤ 0.01, ***P ≤ 0.001, ****P ≤ 0.0001. Graph depicts the mean ± S.E.M. n=X biologically independent cells.

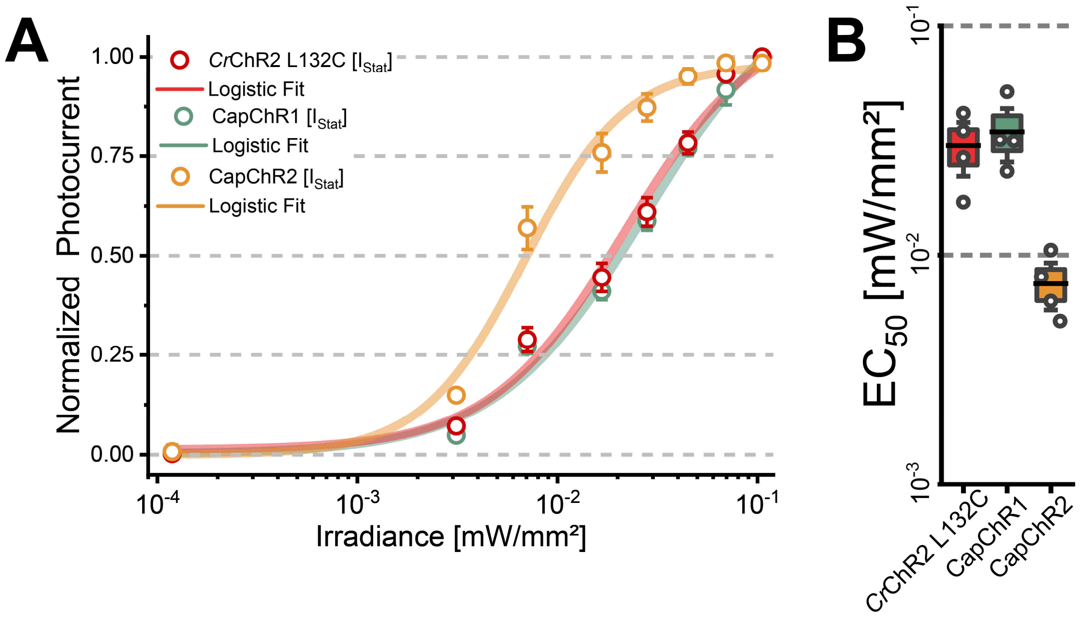

Supplementary Fig. 11: Light sensitivity measurements on ChR2 L132C (red), CapChR1 (green) and CapChR2 (orange). A) Stationary photocurrent as a function of irradiance/light intensity fitted with a logistic function (connecting lines). Dependencies were measured at 140 mM $[NaCl]_e$ and 2 mM $[CaCl_2]_e$ and normalized to the maximal stationary photocurrent. (Mean ± S.E.M , n = 4) B) Estimated half-maximal response values (EC_{50}) obtained from logistic fits (Box middle line: Mean, Box edges: ± S.E.M, Whiskers: ± S.E.M X 1.5, N = 4, dots represent single measurements). n=X biologically independent cells.

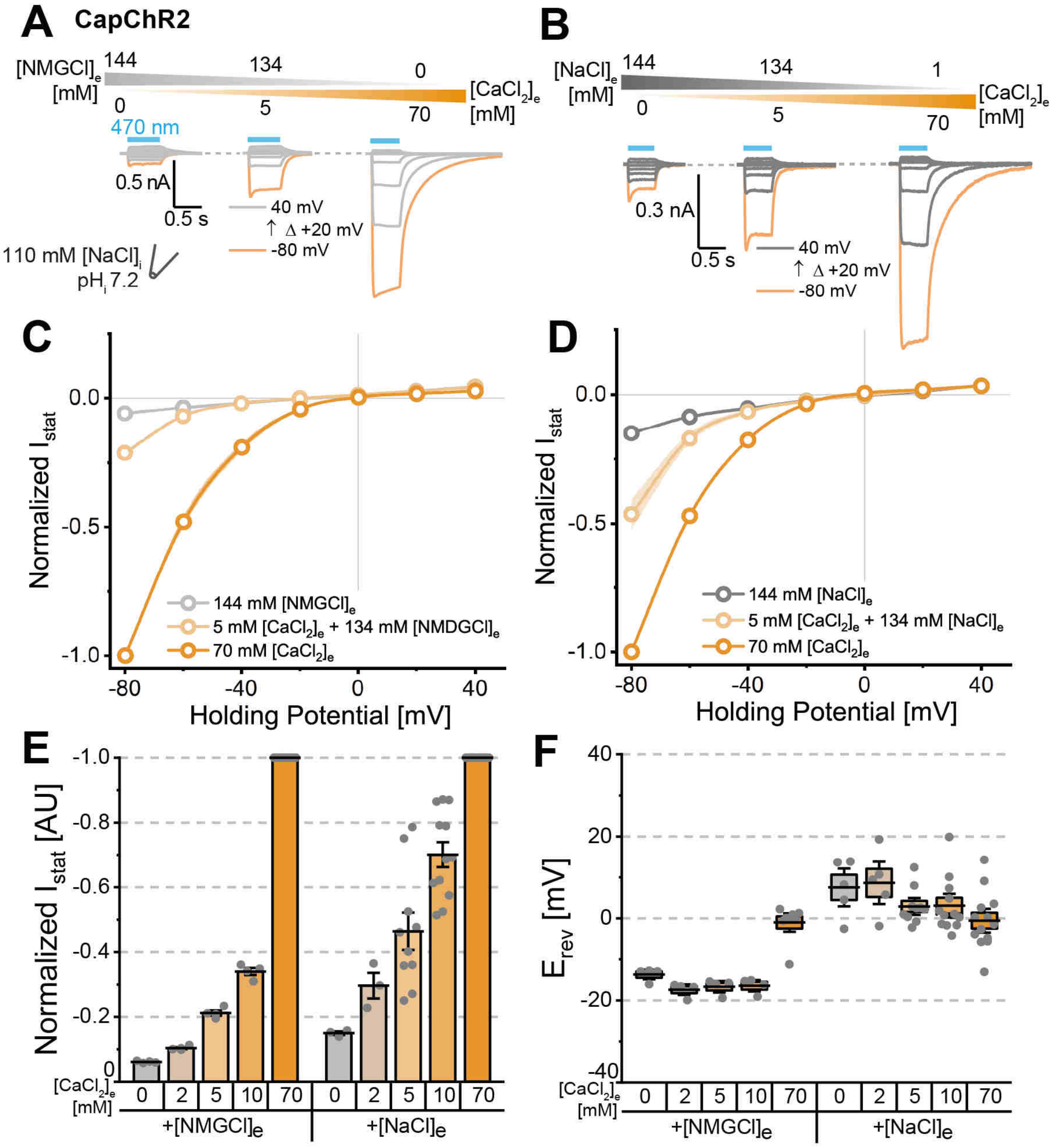

Supplementary Fig. 12: Calcium vs. sodium and calcium vs. proton permeation in CapChR2. A and B) Representative photocurrents traces of CapChR2, recorded from -80 to +40 mV in 20 mV at different A) calcium/sodium or B) calcium/NMGCl concentrations in ND7/23 cells (blue bar: illumination with saturating, 470 nm light; ~1.9 mW/mm²). C and D) I-V relationships of CapChR2 at the designated ionic conditions (Mean ± S.E.M., N = 3-13). Currents normalized to 70 mM $[CaCl_2]_e$. Dots represent mean values and shadows the S.E.M. E) Normalized photocurrents of CapChR2 at -80 mV holding potential and at the denoted $[CaCl_2]_e$ in the presence of sodium or NMGCl. Bars represent the mean ± S.E.M (N = 3-13) and dots represent single measurements. F) Estimated reversal potentials of CapChR2 at the denoted $[CaCl_2]_e$ in the presence of sodium or NMGCl (Box middle line: Mean, Box edges: ± S.E.M, Whiskers: ± S.E.M X 1.5, n = 3-13, dots represent single measurements). n=X biologically independent cells.

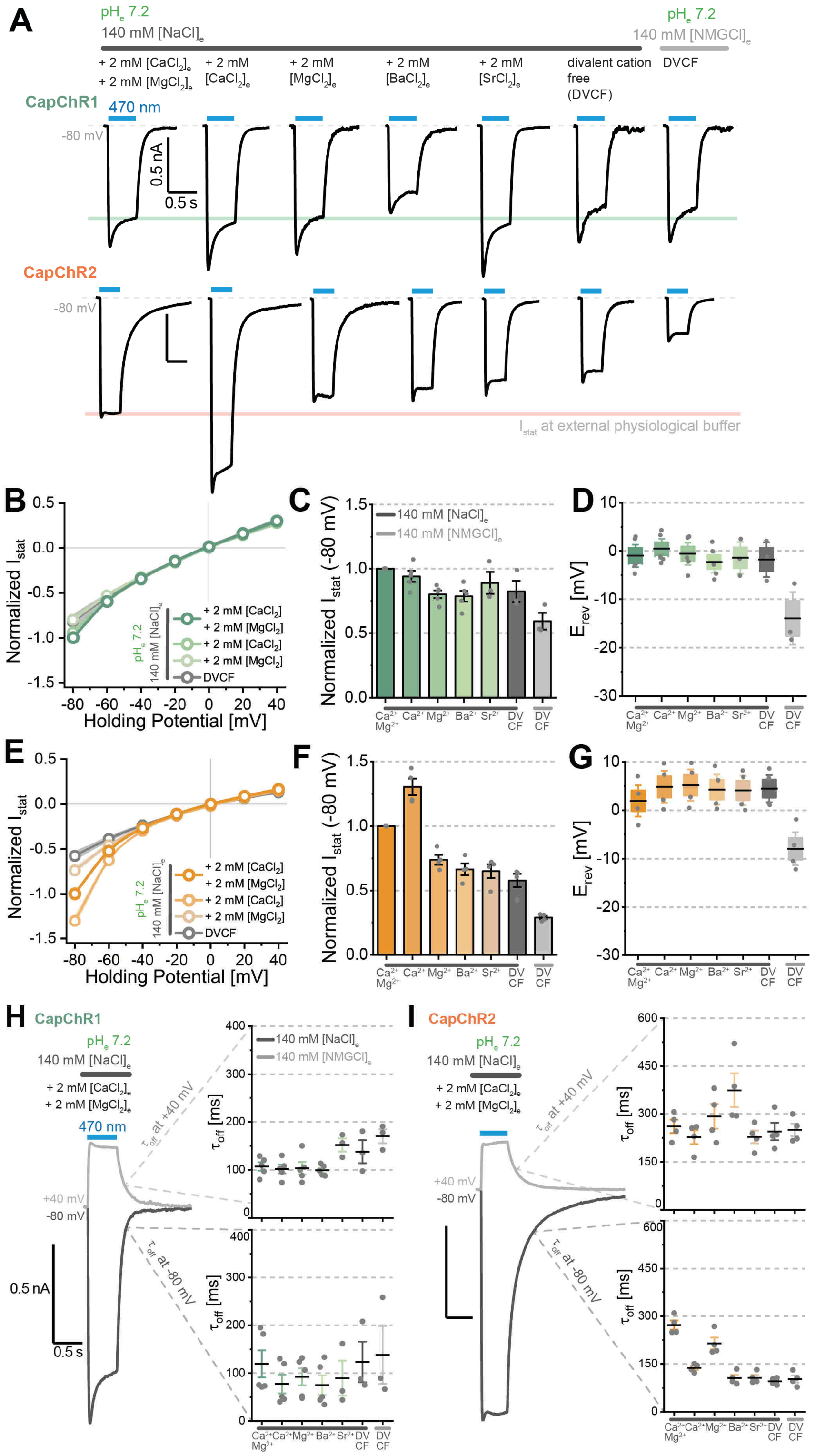

Supplementary Fig. 13: Effect of extracellular divalent cations on CapChR1 (green) and CapChR2 (orange) photocurrents. A) Exemplary photocurrent traces at -80 mV under the denoted buffer conditions and an intracellular buffer containing high [NaCl] and divalent cation free (DVCF, see methods; blue bar: illumination with saturating, 470 nm light; ~1.9 mW/mm²). B and E) I-V relationships for CapChR1 and CapChR2 at the designated ionic conditions (Mean ± S.E.M., n = 4-5). C and F) Normalized Photocurrents in the presence or absence of different divalent cations in the bath solution (Mean ± S.E.M., n = 3-5). D and G) Estimated reversal potentials in the presence or absence of different divalent cations in the bath solution (Box middle line: Mean, Box edges: ± S.E.M, Whiskers: ± S.E.M X 1.5, n = 3-5). H and I) Effect of divalent cations on channel closure rates (τ_{off}). Lines and bars represent the mean ± S.E.M and dots represent single measurements (Mean ± S.E.M., N = 3-5). DVCF: divalent cation free. n=X biologically independent cells.

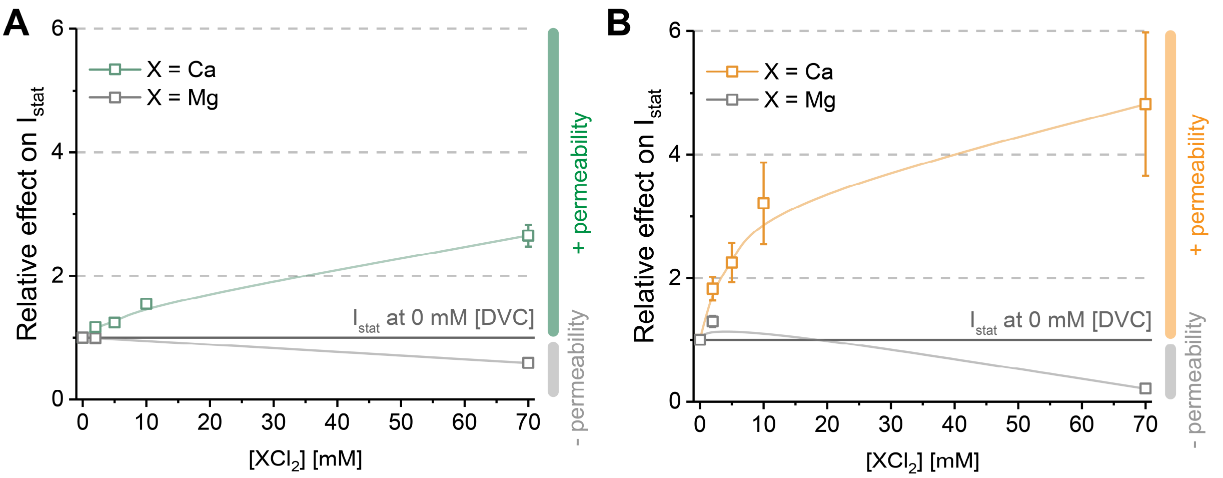

Supplementary Fig. 14: Calcium and Magnesium permeation in CapChR1 (A, green) and CapChR2 (B, orange). Data according to measurements from Figure 3 (Ca^{2+}) and S9 (Mg^{2+}) and S13 (Mg^{2+}/Ca^{2+}). Connecting line is plotted for visual guidance. DVC: Divalent cation. Number of replicates for A): all Ca^{2+} conditions n=6, all Mg^{2+} conditions n=3. Number of replicates for B): all Ca^{2+} conditions n=5, 0 and 2 mM Mg^{2+} conditions n=4; 70 mM Mg^{2+} n=3. Graphs depict the mean ± S.E.M. n=X biologically independent cells.

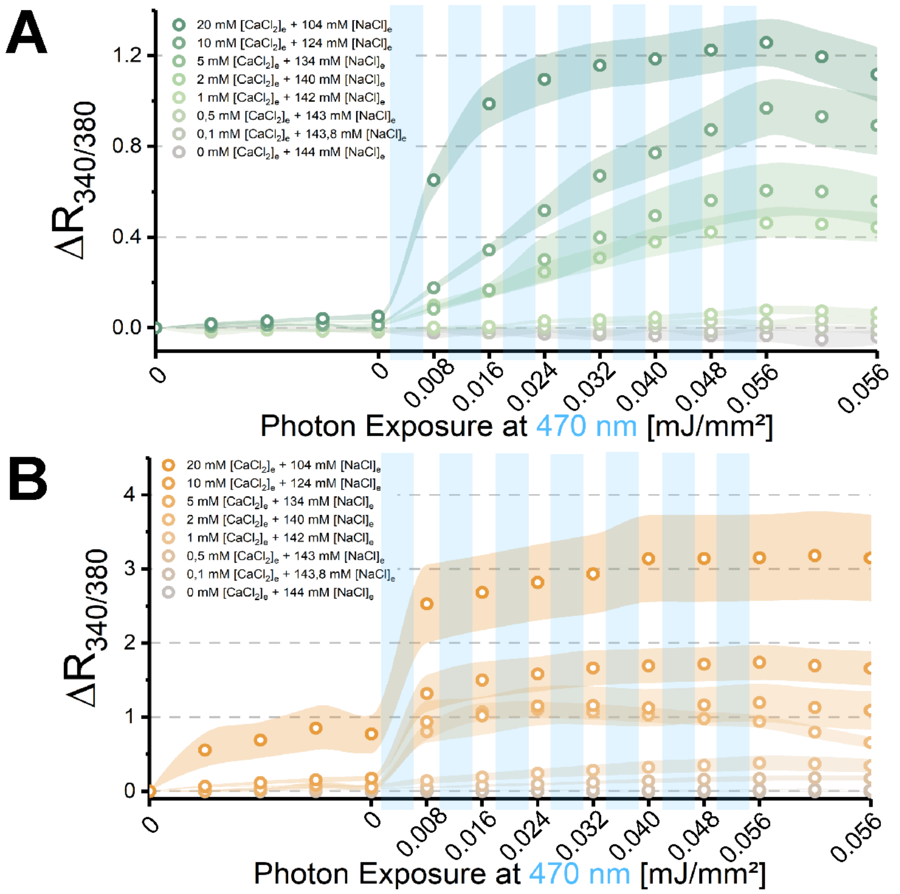

Supplementary Fig. 15: Voltage-clamped calcium imaging on CapChR1 and 2 (complete Datasets from Fig. 4G and H). Illumination: 100 ms, ~0.08 mW/mm², ~0.008 mJ/mm² per $F_{340/380}$ ratio acquisition, saturating illumination for both CapChRs. A and B) Calcium imaging response (ratio of 340/380 nm fluorescence; $R_{340/380}$) for CapChR1 (green) and CapChR2 (orange) at pH 7.2, -80 mV holding potential and differing calcium/sodium ratios. Mean ± S.E.M, n = 6-45 for CapChR1, n = 3-29 for CapChR2. n=X biologically independent cells.

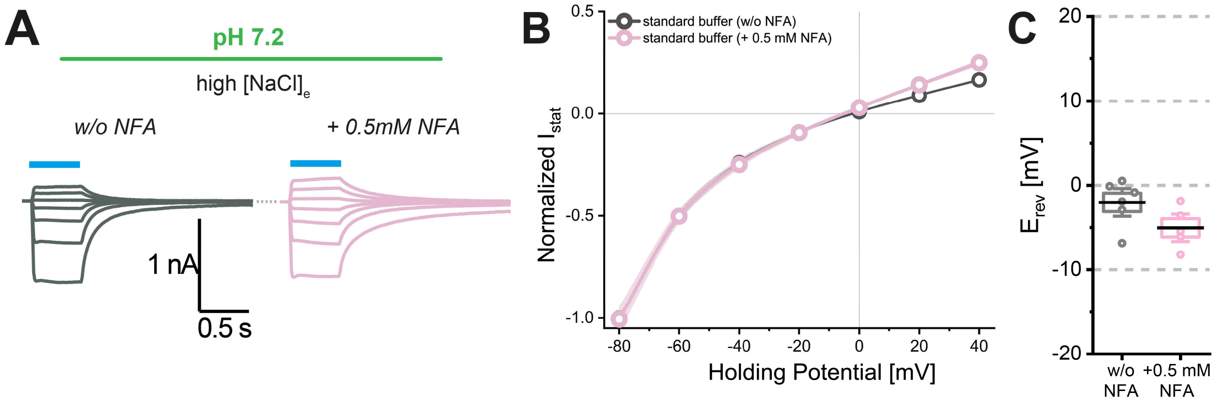

Supplementary Fig. 16: Effects of niflumic acid (NFA) on CapChR2 photocurrents in ND7/23 cells. A) Representative photocurrent traces at 140 mM $[NaCl]_e$ and 2 mM $[CaCl_2]_e$ (standard buffer) with and without (w/o) NFA, recorded from -80 to +40 mV in 20 mV steps in ND7/23 cells. B) Effect of NFA on I-V relationships (Mean ± S.E.M., N = 5-6). Dots represent mean values and shadows the S.E.M. C) Effect of NFA on reversal potentials of CapChR2. Box middle line: Mean, Box edges: ± S.E.M, Whiskers: ± S.E.M X 1.5, N = 5-6, dots represent single measurements. n=X biologically independent cells.

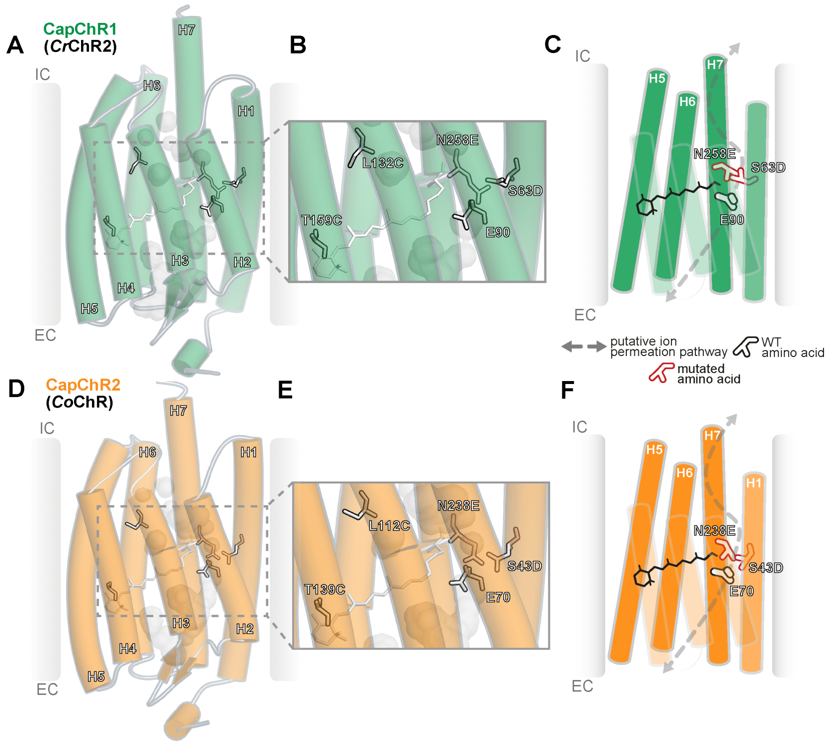

Supplementary Fig. 17: Orientation of residues at the CG of CapChR1 and 2 according to MD simulations (based on structure data, PDB ID: 6EID, http://doi.org/10.2210/pdb6EID/pdb). A) CapChR1 and D) CapChR2 structural models obtained from MD simulations including water densities after 10 ns runtime (grey mesh). B and E) Zoom in on the central gate residues of CapChR1 and CapChR2 respectively. C and F) Proposed orientation of residues and ion permeation according to experimental and MD datasets. EC: extracellular, IC: intracellular.

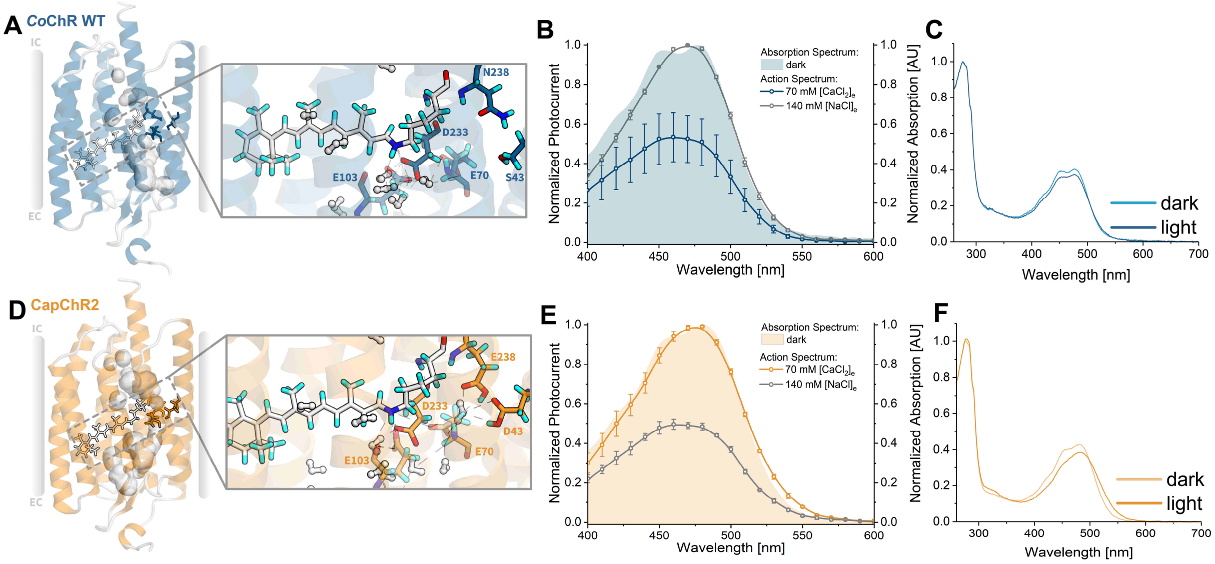

Supplementary Fig. 18: Effect of mutation in the proximity of the Central Gate (CG). A) *Co*ChR WT and D) CapChR2 structural models obtained from MD simulations including water densities after 10 ns runtime (grey mesh; based on structure data, PDB ID: 6EID, http://doi.org/10.2210/pdb6EID/pdb). Denoted amino acids in stick representation close to the central gate and RSB region. Detailed views on the right: CG and RSB region. Dotted lines represent possible interaction within 5 Å of each other. B) and E) action spectra under extracellular high sodium and high calcium conditions (Mean ± S.E.M., n = 3-4) in comparison to the measured absorption spectra (shadows) for *Co*ChR WT (blue) and CapChR2 (orange). C) and F) Absorption spectrum of *Co*ChR WT and CapChR2 respectively before (dark) and after (light) illumination with blue light. AU: arbitrary units, EC: extracellular, IC: intracellular. n=X biologically independent cells.

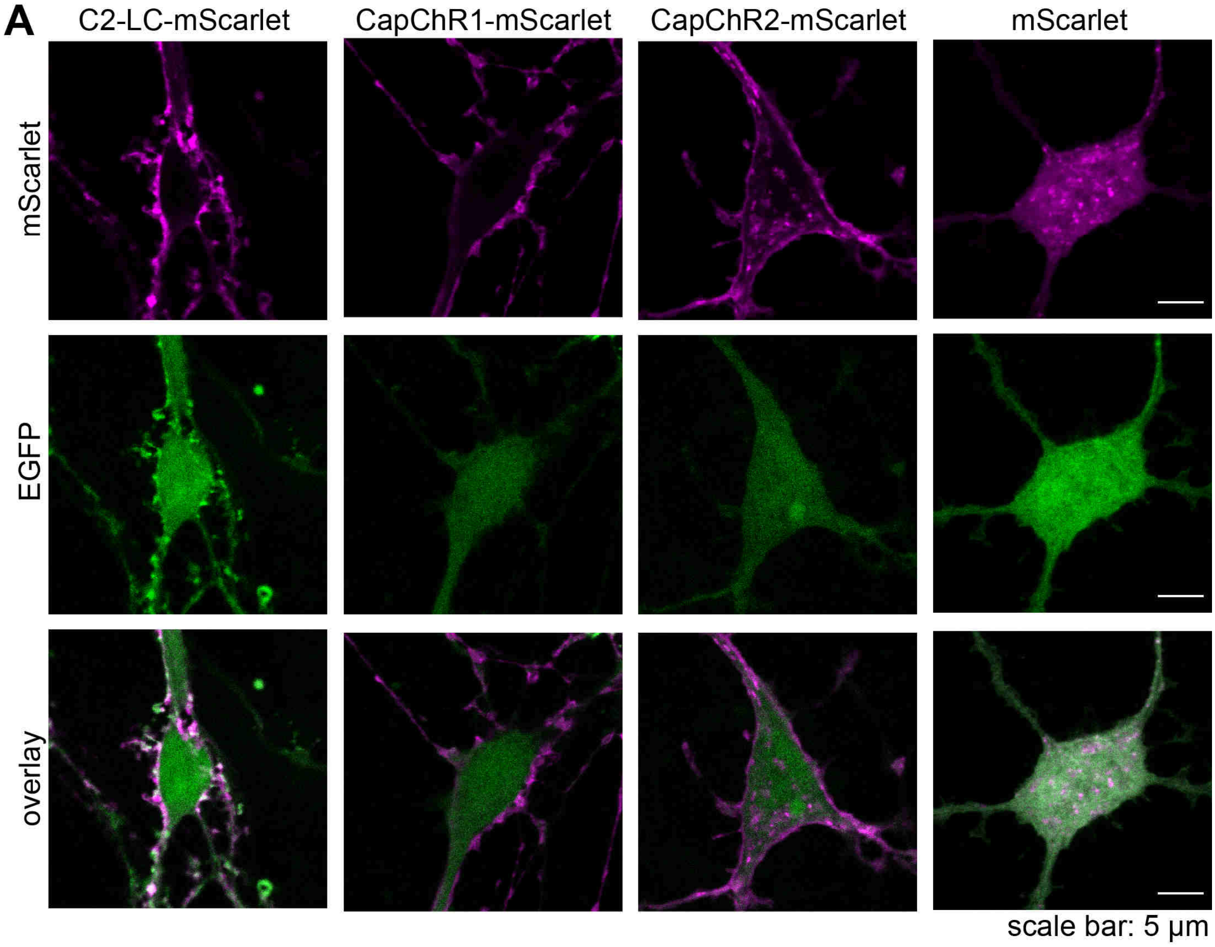

Supplementary Fig. 19: C2-LC, CapChR1 and CapChR2 show efficient and comparable membrane expression in primary neurons. First row (magenta) depicts single plane confocal images of neurons expressing C2-LC-mScarlet (1st column), CapChR1-mScarlet (2nd column), CapChR2-mScarlet (3rd column), or soluble mScarlet (4th column). Neurons also coexpressed a soluble EGFP as a cytosolic volume marker (2nd row). The overlay images (3rd row) illustrate the efficient membrane expression of all three Channelrhodopsins, which strongly contrasts to the cytosolic localization of the soluble mScarlet. Scale bars: 5 µm.

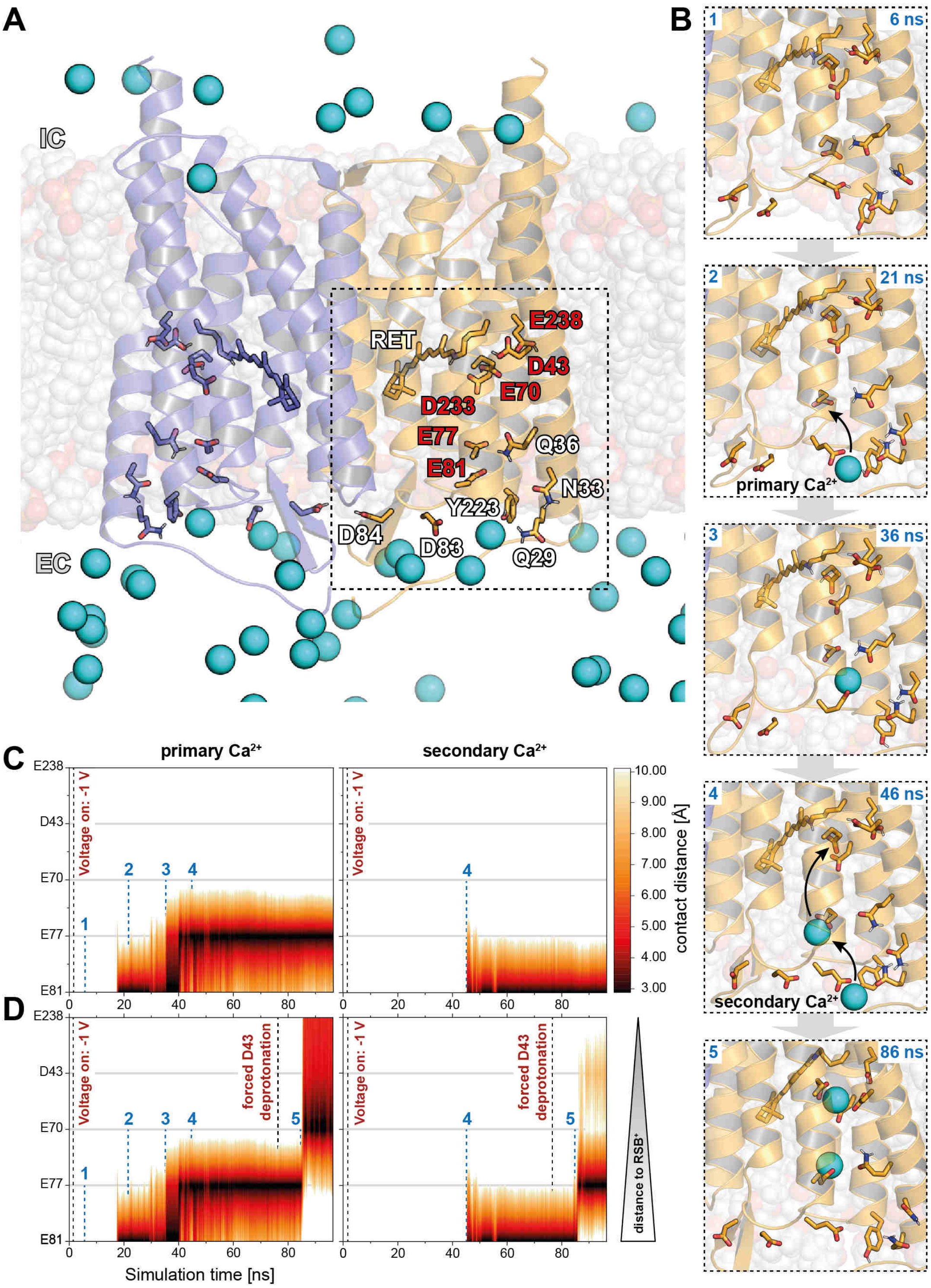

Supplementary Fig. 20: Ca^{2+} uptake simulations of CapChR2 (based on structure data, PDB ID: 6EID, http://doi.org/10.2210/pdb6EID/pdb). A) Initial CapChR2 3-D model with 150 mM [Ca^{2+}], relevant amino acid sidechains shown as licorice, Ca^{2+} binding residues labelled in red and Ca^{2+} shown as cyan spheres. B) Trajectory snapshots from the voltage MD simulations depicting consecutive steps in the Ca^{2+} permeation chain (only relevant Ca^{2+} shown): 1 - Before Ca^{2+} is bound 2 - primary Ca^{2+} bound to E81 3 - primary Ca^{2+} transferred to E77 via side chain rotation into the pore 4 - secondary Ca^{2+} bound to E81 5 - "knock-on" of both Ca^{2+} further into the pore. C) Contact map of primary and secondary Ca^{2+} ions related to pore residues D43, E70, E77, E81 and E238 from initial and D) repeated voltage MD simulation, moment of voltage switch to -1 V highlighted at 1 ns, moment of forced D43 deprotonation at 76 ns, time markings for snapshots 1-5 highlighted at 6, 21, 36, 46 and 86 ns. EC: extracellular, IC: intracellular.
