# Supplementary material for: Calcium-permeable channelrhodopsins for the photocontrol of calcium signalling
Source: Nat Commun. 2022 Dec 21;13:7844. doi: 10.1038/s41467-022-35373-4 (PMC9772239; doi:10.1038/s41467-022-35373-4)
Supplement: Supplementary file 3 — Description of Additional Supplementary Files [file 41467_2022_35373_MOESM3_ESM.pdf]

Description of Additional Supplementary Files

Supplementary Data 1: List of primers used for site-directed mutagenesis.
